# Brain tumor related epilepsy: pathophysiological approaches and rational management of antiseizure medication

**DOI:** 10.1186/s42466-022-00205-9

**Published:** 2022-09-05

**Authors:** Sabine Seidel, Tim Wehner, Dorothea Miller, Jörg Wellmer, Uwe Schlegel, Wenke Grönheit

**Affiliations:** 1 Department of Neurology, University Hospital Knappschaftskrankenhaus, Ruhr University Bochum, In der Schornau 23-25, 44892 Bochum, Germany; 2 Department of Neurosurgery, University Hospital Knappschaftskrankenhaus, Ruhr University Bochum, In der Schornau 23-25, 44892 Bochum, Germany

**Keywords:** Brain tumor related epilepsy, Antiseizure medication, Glioma, Brain metastasis

## Abstract

**Background:**

Brain tumor related epilepsy (BTRE) is a common complication of cerebral tumors and its incidence is highly dependent on the type of tumor, ranging from 10–15% in brain metastases to > 80% in low grade gliomas. Clinical management is challenging and has to take into account aspects beyond the treatment of non-tumoral epilepsy.

**Main body:**

Increasing knowledge about the pathophysiology of BTRE, particularly on glutamatergic mechanisms of oncogenesis and epileptogenesis, might influence management of anti-tumor and BTRE treatment in the future. The first seizure implies the diagnosis of epilepsy in patients with brain tumors. Due to the lack of prospective randomized trials in BTRE, general recommendations for focal epilepsies currently apply concerning the initiation of antiseizure medication (ASM). Non-enzyme inducing ASM is preferable. Prospective trials are needed to evaluate, if AMPA inhibitors like perampanel possess anti-tumor effects. ASM withdrawal has to be weighed very carefully against the risk of seizure recurrence, but can be achievable in selected patients. Permission to drive is possible for some patients with BTRE under well-defined conditions, but requires thorough neurological, radiological, ophthalmological and neuropsychological examination.

**Conclusion:**

An evolving knowledge on pathophysiology of BTRE might influence future therapy. Randomized trials on ASM in BTRE with reliable endpoints are needed. Management of withdrawal of ASMs and permission to drive demands thorough diagnostic as well as neurooncological and epileptological expertise.

## Background

Primary brain tumors account for about 1.6% of all cancers [1]. Incidence of primary malignant and non-malignant brain tumors was 23.79 per 100,000 inhabitants per year in the U.S. for the years 2013–2017 (data from the central brain tumor registry of the United States, CBTRUS) [2]. Brain metastasis occur in about 25% of solid tumors [3]. The risk of brain tumor related epilepsy (BTRE) is highly dependent on tumor histology. Patients with diffuse low-grade gliomas suffer from BTRE in > 80% of cases [4, 5], while 62–68% patients with glioblastomas [6, 7] and 40–47% of patients with meningiomas [8, 9] have seizures. In patients with brain metastases the frequency of BTRE is 10–15% [10, 11].

Frontal, parietal and temporal localization of the brain tumor is reportedly associated with a higher risk of seizures compared to occipital localization [12, 13]. In patients with brain metastases, > 4 metastases, high risk location of metastases (defined as frontal, parietal, temporal, or occipital cortex) and melanoma as the primary tumor are risk factors for the occurrence of BTRE [11]. BTRE has a negative impact on quality of life [14]. In this review, an overview of the pathophysiology of BTRE is given. Rational antiseizure medication (ASM) after the first seizure, in case of persistent seizures and in refractory BTRE is discussed (taking into account possible anti-tumor effects of ASM). Further, the duration of ASM and clinical management concerning fitness to drive are addressed in this article. We do not discuss the role of tumor specific treatment (surgery, radiotherapy, chemotherapy) for the reduction of seizure frequency. We neither address the topic of prophylactic ASM in seizure free brain tumor patients.

## Main text

### Pathophysiology of BTRE: what the clinician needs to know

**Fig. 1:**
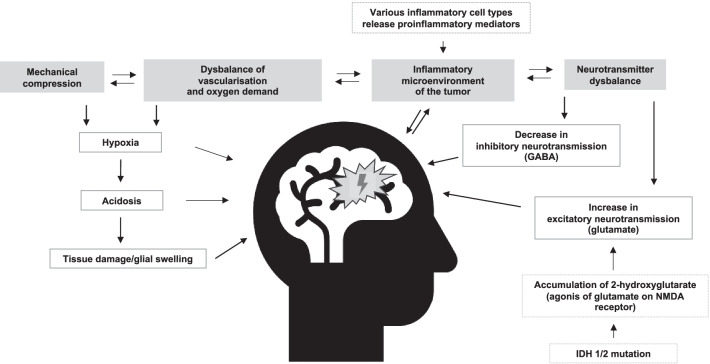
Overview of mechanisms driving brain tumor related epilepsy (simplified)

Multiple mechanisms driving the pathophysiology of BTRE have been described. Mechanical compression, imbalance of vascularization and oxygen demand of the tumor, inflammatory processes and neurotransmitter dysbalance play the major roles in epileptogenesis in brain tumor patients (Fig. 1).

**Fig. 2:**
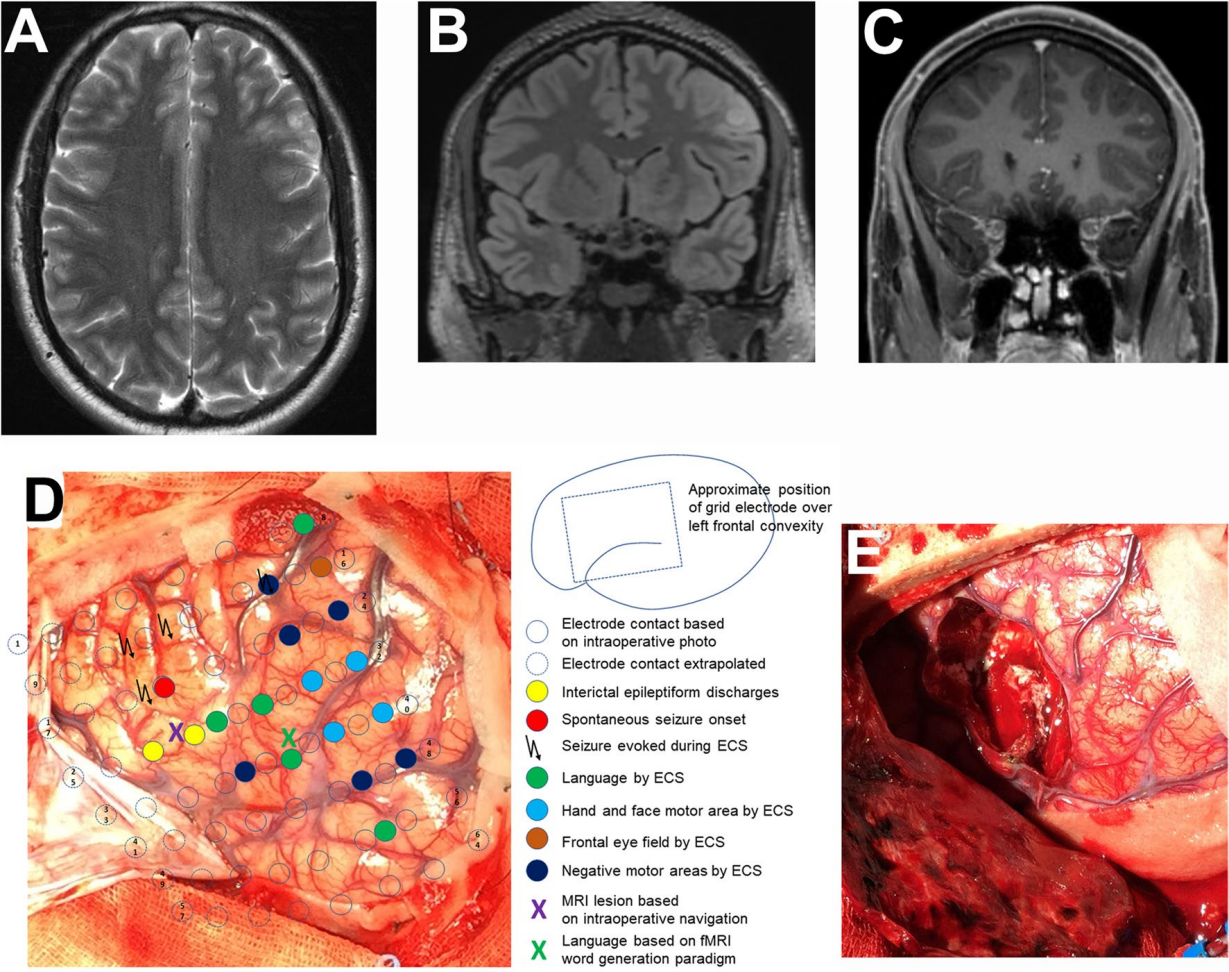
Brain tumor in the left middle frontal gyrus with cortical and subcortical T2/FLAIR hyperintense signal (**A**, **B**) and partial Gadolinium enhancement (**C**) in a 24-year-old male patient. **A** Axial T2, **B** coronal FLAIR, **C** coronal T1 with Gadolinium, **D** intraoperative situs with superimposed results from extraoperative subdural electrode recording and direct cortical stimulation, **E** intraoperative situs following resection of the tumor and adjacent epileptogenic area (histology: angiocentric glioma WHO grade 1). Figure modifed from Wehner et al. [85]

Changes in neurotransmitter balance lead to epileptogenicity of the tumor itself as well as of the tissue adjacent to the lesion, which means that the “epileptogenic zone” [15] includes the peritumoral tissue. Thus, even a complete resection of a brain tumor does not necessarily mean that the epileptogenic zone has also been removed, since a relevant part of seizures in BTRE is generated by the surrounding tissue (Fig. 2).

Mechanical compression may induce ischemia and metabolic changes with subsequent blood brain barrier disruption leading to a higher risk of seizures. The vascular supply of brain tumors is often unbalanced and insufficient for the oxygen demand of the tumor and the peritumoral region, leading to hypoxia of the tissue and subsequently to acidosis, glial swelling and tissue damage. These factors contribute to epileptogenesis in brain tumor patients [16].

Inflammatory processes play an important role in oncogenesis and malignant progression of brain tumors through various mechanisms [17]. On the other hand, an interplay of inflammation and occurrence of seizures has been repeatedly reported. While brain inflammation can induce seizures, recurrent or ongoing seizure activity can maintain chronic inflammation [18, 19]. In this context, it is of note that immune checkpoint inhibitors, which reinforce antitumor activity of the immune system, might also lead to a higher risk of status epilepticus. In a retrospective analysis an increasing number of status epilepticus in brain metastasis patients had been observed since the approval and increasing use of immune checkpoint inhibitors [20].

The influence of neurotransmitter imbalance on epileptogenesis in BTRE, in particular a decrease in inhibitory GABA-ergic neurotransmission and an increase in excitatory glutamatergic synaptic input, has been shown in several preclinical and clinical studies [21]. Further, within the last decade, several studies supporting an overlap of mechanisms involved in oncogenesis as well as in epileptogenesis have been published [22, 23]. One fundamental finding was the ability of glioma and breast cancer brain metastasis cells to integrate into neuronal circuits. Gliomas are able to form microtubes, which are thin tubes with membranes, that look like axonal and dendritic growth of developing neurons. On the surface of these microtubes functional synapses between neurons and glioma cells (*neurogliomal synapses*) were found that communicate via postsynaptic currents mediated by α-Amino-3-hydroxy-5-methylisoxazol-4-propionsäure (AMPA) glutamate receptors [24, 25]. Further, breast cancer brain metastasis [26] and glioma cells [24] were able to form tripartite glutamatergic synapses of cancer cells and pre- and postsynaptic neuron. Perturbation of the glutamatergic pathways in functional *neurogliomal synapses* as well as perturbation of the indirect perisynaptic communication of neurons and breast cancer cells led to a reduction of invasive growth and slower proliferation of the tumor cells in preclinical studies. This implies that seizure activity can induce brain tumor proliferation [24, 26]. Conversely, glioma progression can lead to neuronal hyperexcitability, which is a typical feature of epilepsy [27]. In line with these findings, median overall survival of patients with brain metastases or glioblastomas and status epilepticus was inferior to that of patients with the same diseases without status epilepticus in a retrospective analysis on 1792 patients (742 meningiomas, 249 glioblastomas, 801 brain metastases) [28]. In this study, the OS of brain metastasis patients with BTRE was significantly inferior to that of patients without epilepsy. An influence of BTRE on overall survival was not found for patients with glioblastoma or meningioma. It is of note that for patients with meningiomas, synapses between neurons and tumor cells have not been not reported [24].

The interplay of neurons and brain tumor cells implies on the one hand a possible role of seizures to promote brain tumor progression and on the other hand potential therapeutic targets to treat both tumor and BTRE [27]. One of the possible therapeutic targets is an inhibition of the AMPA signaling via α-Amino-3-hydroxy-5-methylisoxazol-4-propionsäure receptor (AMPAR) inhibitors. While talampanel, a non-competitive AMPAR inhibitor, has not found its way into clinical practice due to its unfavorable pharmacokinetic profile [29], perampanel, also a non-competitive AMPAR inhibitor, is an approved and increasingly used medication in focal epilepsies. Randomized placebo controlled clinical trials on the use of perampanel in brain tumor patients have not been published to date, but are planned for the foreseeable future. A different approach of targeting glutamatergic mechanisms might be blocking of the overexpression of the cystine-glutamate transporter (xCT), which is common in glioma cells. The xCT exchanges intracellular glutamate for extracellular cystine, thus upregulating both, extracellular glutamate and intracellular cystine, which is used by glioma cells for production of the antioxidant glutathione. Sulfasalazine is a drug able to block the overexpression of xCT, however, data evaluating the use of this drug in clinical practice is lacking [30]. Further, drugs that target the receptor PPAR-λ (e.g., glitazones) are able to induce amino acid transporters on astrocyte membranes, which increases reuptake of glutamate from the extracellular space. These drugs have also shown potential to diminish tumor growth in the preclinical setting [30].

Aberrant GABA signaling leads to accumulation of chloride in glioma cells, which influences both epileptogenicity and oncogenesis. Dysfunction of the mammalian target of rapamycin (mTOR) pathway can lead to modification of glutamate and GABA signaling. Both mechanisms might be potential targets for concomitant treatment of the tumor and of BTRE [30].

Of the molecular markers, which are of increasing importance for treatment decisions in glioma patients, isocitrate-dehydrogenase isoenzyme 1 or 2 (IDH 1/2) is to date the only marker that has been reported to be associated with a higher incidence of seizures. Several clinical studies have shown that an IDH 1/2 mutation confers a risk for preoperative [31, 32] and perioperative [33] seizures in brain tumors. The IDH 1/2 mutation leads to a loss of the ability of the IDH to catalyze the conversion of isocitrate to α-ketoglutarate and further results in a gain of the ability of the IDH to catalyze the reduction of α-ketoglutarate to 2-hydroxyglutarate [34]. This mechanism leads to an accumulation of 2-hydroxyglutarate, which has structural similarity to glutamate and is able to activate the NMDA receptor, therefore acting as a glutamate agonist. A higher glutamate level results in a higher seizure probability. This agonistic mechanism explains the epileptogenic potential of the IDH mutations [35]. While IDH mutations were associated with the occurrence of seizures in a cohort of 442 glioma patients with preoperative seizures in China, p53 expression, ATRX loss, MGMT gene promotor methylation, TERT promoter mutation and 1p/19q co-deletion status were not associated with a higher risk of seizures in this retrospective analysis [36].

### Management of BTRE after first seizure

In brain tumor patients, the occurrence of one seizure implies the diagnosis of epilepsy [37]. As brain tumors may grow continuously over time (according to the tumor type), the concept of acute symptomatic seizures does not apply for brain tumor patients. Thus, ASM are usually initiated after the first seizure according to the general recommendations of the International League against Epilepsy (ILAE). Histology, grading, location and molecular markers of the tumor currently do not play a role for the choice of ASM [38]. For valproic acid (VPA) [7, 39–41] and levetiracetam [42, 43], several clinical studies had suggested an antineoplastic effect in patients with glioblastomas, however, a combined metanalysis of four prospective trials did not confirm this effect [44]. Small series on the antiseizure effectivity of perampanel in BTRE have been published [45], clinical studies on its influence on oncogenesis and tumor proliferation are planned for the future. Thus, ASMs in BTRE at present should be chosen based on their pharmacokinetic and pharmacodynamic properties, tolerability and side effects and not for a possible anti-tumor effect. Consensus exists that non-enzyme inducing ASMs should be preferred to enzyme inducing ASMs to avoid interference with antineoplastic and other drugs (e.g., dexamethasone) [38]. Non-enzyme inducing drugs commonly used as monotherapy are: lacosamide (LCM), lamotrigine (LTG), levetiracetam (LEV), topiramate (TPM), valproic acid (VPA) and zonisamide (ZNS) [46]. Larger series (> 25 patients) on GBP and ZNS use in BTRE are lacking. For the other ASMs an overview of studies in BTRE that reported results for each drug is given in Table 1.

**Table 1:** Overview of studies on lacosamide, lamotrigine, levetiracetam, topiramate and valproic acid in brain tumor related epilepsy (studies that included ≥ 25 patients on adults with ≥ 3 months observation time)

Article	No. of patients^a^	Study design	Type of tumor	Mono-/polytherapy	Follow-up (months)	Outcome/main endpoints
*Lacosamid*						
Maschio et al. (2017) [57]	25	Pros	Glioma “High-grade” n = 12 “Low-grade” n = 13	Poly	5.8 (mean)	Seizure free at final follow-up: 28% Reduction of seizures ≥ 50%: 48% (additional to seizure free patients)
Mo et al. (2022) [63]	132	Retro	Primary brain tumor	Mono	Follow up at 3 and 6 months	3-months seizure-free: 64.4% 6-months seizure-free: 55%
Van Opijnen et al. (2021)^b^ [48]	78	Retro	Glioma Grade 2 (n = 31) Grade 3 (n = 11) Grade 4 (n = 36)	Poly (71%)	Maximum of 36 months	12-months cumulative incidence of treatment failure: 30% 12-months cumulative incidences of treatment failure uncontrolled seizures: 11% 12-months cumulative incidences of treatment failure due to adverse events: 19%
Ruda et al. (2017) [58]	71	Pros	Glioma Grade 2 (n = 26) Grade 3 (n = 20) Grade 4 (n = 25)	Poly	Follow-up at 3, 6, 9 months	3-, 6- and 9-months seizure reduction ≥ 50%: 74.6, 76.0, 86.2% (including seizure free patients) 3-, 6- and 9-months seizure free: 42.2, 43.0, 50%
Ruda et al. (2020) [59]	93	Pros	Glioma “Low-grade” (n = 84) Grade 3 (n = 1) Suspected glioma (n = 3) Meningeoma (n = 3) Other (n = 2)	Poly	6 months observation	6-months seizure reduction ≥ 50%: 76.7% 6-months improvement of Patient’s Global Impression of Change (PGIC): 64.5% 6-months seizure-free: 34.9%
Saria et al. (2013) [60]	70	Retro	Glioma Grade 2 (n = 25) Grade 3 (n = 12) Grade 4 (n = 28) Meningeoma (n = 3) Other (n = 2)	Poly	6.2 (median)	Decrease in seizures: 66% 6-months seizure reduction ≥ 50%: 54% No reported toxicities: 77%
Sepulveda-Sanchez et al. (2016) [61]	39	Retro	Primary brain tumor (n = 31) Metastasis (n = 7) Not reported (n = 1)	Poly	Follow-up at 3 and 6 months	6-months reduction of seizure frequency from 26.4 (mean) to 9.4 (mean) Adverse event: 12%
Villanueva et al. (2016) [62]	105	Retro	“Astrocytoma” (n = 42) Glioblastoma (n = 13) Brain metastasis (n = 11) Meningioma (n = 11) Oligodendroglioma (n = 7) Ganglioglioma (n = 6) Oligoastrocytoma (n = 5) DNET (n = 3) Other (n = 4)	Poly	6 months observation	6 months seizure-free: 30.8% 6-months seizure reduction ≥ 50%: 66.3% (including seizure free patients) Adverse events: 41.9%
*Lamotrigin*						
Van Opijnen et al. 2021^b^ [48]	61	Retro	Glioma Grade 2 (n = 31) Grade 3 (n = 13) Grade 4 (n = 17)	Poly (66%)	Maximum of 36 months	12 months cumulative incidence of treatment failure: 38% 12 months cumulative incidences of treatment failure due to uncontrolled seizures: 18% 12 months cumulative incidences of treatment failure due to adverse events: 17%
*Levetiracetam*						
De Groot et al. (2011) [49]	40 (n = 34 evaluable)	Pros	Glioma Grade 2 (n = 7) Grade 3 (n = 12) Grade 4 (n = 15)	Mono	6 months observation	6-months seizure free: 59% 6-months seizure reduction ≥ 50%: 74%
Kerkhof et al. (2013)^d^ [7]	36	Retro	Glioblastoma (n = 36)	Mono	9 (median)	Seizure free at the end of follow-up (minimum of 6 months): 69.5%
Maschio et al. (2011) [50]	29	Pros	Glioma Grade 2 (n = 6) Grade 3 (n = 10) Grade 4 (n = 9) Meningeoma (n = 2) Other (n = 2)	Mono		12-months seizure freedom for n = 15 patients who reached this endpoint: 93.3% 12-months ≥ 50% seizure reduction: 6.7% (responder rate 100%)
Rosati et al. (2010) [52]	82	Pros	Glioma: Grade 1/2 (n = 13) Grade 3 (n = 15) Grade 4 (n = 54)	Mono	13.1 (mean)	Seizure free with monotherapy levetiracetam at last follow up: 89%
Rossetti et al. (2013) [53]	25	Pros	Glioma Grade 3 or 4 (n = 17) No further details	Mono (n = 9) Poly (n = 14)	12 months observation	Composite endpoint (discontinuation of the study drug, add-on of a further ASM, ≥ 2 seizures with impaired consciousness) during 1 year follow-up: 36% Discontinuation due to side effects: 24%
Van der Meer et al. (2020)^c^ [55]	429	Retro	Glioma, grade 2–4 Grade 2 (n = 108) Grade 3 (n = 44) Grade 4 (n = 277)	Mono	86.2 (median)	Treatment failure for any reason within 36-months follow-up: 40% Treatment failure because of AE within 36-months follow-up: 16% Treatment failure because of uncontrolled seizures within 36-months follow-up: 19%
Wagner et al. (2003) [54]	26	Pros	Glioma Grade 3 and 4 (n = 18) Grade 2 (n = 8)	Poly (n = 25) Mono (n = 1)	9.3 (median)	Seizure free 38% 6-months ≥ 50% seizure reduction: 35%
*Topiramat*						
Maschio et al. (2007) [84]	47 (45 evaluable)	Pros	Glioma Grade 4 (n = 8) Grade 3 (n = 20) “Low grade” (n = 13) Meningeoma (n = 4) Metastasis (n = 2)	Mono (n = 33) Poly (n = 14)	16.5 (mean)	Seizure free: 55.6%, ≥ 50% seizure reduction: 20% Discontinued TPM for severe side effects: 6.4%
*Valproic acid*						
Van der Meer et al. (2020)^c^ [55]	429	Retro	Glioma Grade 2 (n = 105) Grade 3 (n = 44) Grade 4 (n = 280)	Mono	86.2 (median)	Treatment failure for any reason within 36-months follow-up: 56% Treatment failure because of AE within 36-months follow-up: 32% Treatment failure because of uncontrolled seizures within 36-months follow-up: 17%
Kerkhof et al. (2013)^d^ [7]	36	Retro	Glioblastoma (n = 36)	Mono	9 (median)	Seizure free at the end of follow-up (minimum of 6 months): 77.8%

^a^Number patients in the study treated with the respective ASM

^b^Retrospective study comparing n = 61 patients with lamotrigine and n = 78 with lacosamide

^c^Retrospective observational study with matched groups of n = 429 patients each with levetiracetam and valproic acid

^d^Retrospective study on 291 patients with glioblastoma treated with levetiracetam or valproic acid monotherapy or polytherapy of both, efficacy of AED therapy was calculated only for patients who had a minimum follow-up period of 6 months

*AE* adverse event, *ASM* antiseizure medication, *BTRE* brain tumor related epilepsy

The trials reported heterogenous endpoints, most trials had included a variety of different tumor types and investigated variable ASM combinations additional to the study drug, partly due to drug approval only as add-on treatment at the time of the study. Hence, in general, recommendations for ASM choice in BTRE are currently based on therapy recommendations for focal epilepsies. LEV or LTG are valuable treatment options in focal epilepsies [46]. Recently the single-blind SANAD II study (in which, however, existence of a brain tumor had been an exclusion criteria) demonstrated superiority of lamotrigine compared to levetiracetam in focal epilepsies. In particular, the rate of treatment failures due to adverse reactions had been higher with levetiracetam than with lamotrigine [47]. If these results are transferable for brain tumor patients is a currently unresolved question. It has to be taken into account that while LTG itself is not an enzyme inducing drug, its metabolism is influenced by medications that induce the cytochrome P450-3A4 system. The number of studies on lamotrigine in BTRE is very limited. In one retrospective study, the 12-months cumulative incidence of treatment failure had been 38% and the incidence of adverse events 17% with a second line lamotrigine monotherapy (which had been comparable to the alternative treatment with LCM in this study) [48]. Besides potential interaction with anti-tumor medication, disadvantages of LTG are its availability only as an oral formulation and the slow titration at initiation of the medication. Both aspects can be especially relevant in patients with brain tumors in case of reduced consciousness, dysphagia and/or need of immediate seizure control.

Several studies have demonstrated that a medication with LEV is efficacious in BTRE [7, 49–54]. In contrast to lamotrigine, LEV can be applied intravenously, fast titration is possible and virtually no pharmacokinetic interaction has been reported. In a retrospective observational study on two matched groups of 429 patients the cumulative incidence of treatment failure had been lower in patients treated with LEV monotherapy than with VPA monotherapy. While the rate of adverse events was similar in both groups, LEV was more efficacious in seizure control [55]. However, a medication with LEV can lead to relevant neuropsychiatric side effects, particularly in patients with frontal tumors [56].

Another therapeutic option for treatment of BTRE is LCM. An intravenous application is available, titration is fast, the rate of neuropsychiatric side effects is low and there are very few interactions with other drugs. It has shown efficacy in BTRE in several trials, mostly as add-on treatment [57–62]. Recently, a retrospective analysis on LCM as monotherapy in 132 primary brain tumor patients reported seizure freedom in 64.4% of patients at 3 months and 55% at 6 months [63]_._ It is of note that before initiation of LCM an atrioventricular block of second or higher degree has to be excluded via electrocardiogram. A retrospective study in glioma patients, in which LTG (n = 61 patients) and LCM (n = 78 patients) were compared reported similar efficacy and a similar incidence of treatment failure due to adverse events for both ASMs [48].

In addition to the typical side effects of the individual ASMs, potentially beneficial concomitant effects of the medication (e.g., anxiolysis, mood stabilization, sedation) should also be considered when selecting the appropriate ASM.

Beyond choosing an effective ASM with few or no interaction with other medication, relevant criteria for the choice of ASM in patients with BTRE are (1) potentially beneficial side effects, (2) dosage form (oral, intravenous) and (3) avoidance of impairing side effects.

### Management of persistent seizures

In case of remanifestation of seizures in brain tumor patients after former seizure control, tumor progression has to be excluded. Particularly in high grade gliomas recurrent seizures can be a sign of progression [64]. In case of tumor progression in combination with (or diagnosed because of) remanifestation of seizures, the goal of tumor specific treatment can be both: the control of tumor growth and of seizure frequency.

There is no sufficient data in BTRE to determine if an alternative ASM monotherapy or a polytherapy should be preferred in patients who do not become seizure free with their first monotherapy, neither is one ASM superior to others if applied as a second medication [38].

Perampanel and brivaracetam are two more recently approved non enzyme inducing treatment options often used as add-on treatment in focal epilepsies and have shown potential in smaller series in BTRE. In a pilot study on 26 glioma, meningioma and brain metastasis patients (21 patients could be evaluated at 6 months) treated with perampanel as add-on treatment, eight of 21 patients (31%) were seizure free at 6 months and 20 of 21 patients (95%) had a ≥ 50% seizure reduction [45]. In another study on perampanel in BTRE, 21 of 36 patients were evaluable for response to perampanel at 12-months follow-up. Of 21 patients seven patients were seizure free (33%) and 12 more patients had a seizure reduction ≥ 50% (≥ 50% seizure free rate 90%, 19/21 patients) [65]. For Brivaracetam a reduction of the seizure frequency from 7 per month to 2 per month was reported in a retrospective analysis on 33 primary brain tumor patients [66].

If cenobamate, a recently approved drug for focal epilepsies with promising results in two randomized placebo-controlled trials, might be able to play a therapeutic role for BTRE will have to be determined in the future [67, 68]. In the approval studies patients with potentially progressive causes of epilepsy were not eligible for inclusion.

As a practical approach, combination therapy is preferable if the first ASM has reduced seizure frequency, but has failed to control epilepsy completely. If the first ASM did not have any effect on seizure frequency, a second monotherapy should be initiated. The general recommendation to prefer non-enzyme inducing drugs whenever possible also applies for the remanifestation of seizures or for refractory seizures. A combination of two ASMs with the same mechanism of action is often associated with increased side effects.

### Treatment of refractory seizures and status epilepticus

About 60% of patients with brain tumors do not become seizure free with the first ASM and of those patients, only 40% eventually become seizure-free with a second line monotherapy or polytherapy [69].

For patients with refractory BTRE (who usually require an antiseizure polytherapy, often including > 2 drugs), weighing side effects of ASM against a lack of seizure control is very important, especially at an advanced disease stage, when the therapeutic goal shifts from tumor control to mere symptom control. A retrospective study that included 100 patients with BTRE reported significantly more side effects, cognitive deficits and a lower quality of life in patients with antiseizure polytherapy, while the number of seizures was not related to quality of life [14].

In adults, 7% of status epilepticus (SE) are due to brain tumors and SE in BTRE is associated with significant mortality [70]. In their systematic review, Arik et al. found no evidence for the superiority of a particular ASM in tumor-related SE. Further data regarding the specificities in duration, prognosis and efficacy of ASM in tumor-related SE is needed. At present, treatment recommendations for SE in BTRE are based on treatment recommendations for SE in all epilepsies [71].

Especially at the advanced tumor stage, quality of life impairment caused by medication must be critically examined and individually weighed against the impairment by recurrent seizures. In very few patients with refractory seizures or status epilepticus, palliative tumor/epilepsy surgery may be considered as a treatment option for seizure reduction after careful individual evaluation in centers with combined neurooncology and epilepsy surgery expertise.

### Adjustment of therapy for dysphagia in BTRE patients

Dysphagia is a frequently observed symptom in brain tumor patients. While some patients have swallowing difficulties in an early disease stage, dysphagia is a symptom more often observed in an advanced tumor stage. It can be caused by the tumor itself, by recurrent seizures or SE. Therefore, the dosage forms available for the various preparations should be taken into account for the choice of ASM in individual treatment situations. Many ASMs are available as oral liquids, which often are easier to swallow. For some of the new anticonvulsants (e.g., LEV, LCM), clinical experience in off-label subcutaneous use has been reported and has shown practical applicability in the palliative setting [72]. LEV can be applied as a continuous infusion via a syringe driver or as intermittent boli diluted in 100 ml 0.9% sodium chloride every 12 h over 30 min [73]. Case reports for subcutaneous use of LCM [74] (as an undiluted solution over 10 min) and BRV [75] (as a continuous infusion via syringe driver) have been published before. The oral to subcutaneous conversion rate was 1:1 for LEV, LCM and BRV. Proactively changing the dosage form in patients with dysphagia before a decrease in serum levels of ASM occurs might avoid seizures and SE.

### Withdrawal of ASM

There is limited data on a possible cessation or withdrawal of ASM in brain tumor patients with epilepsy. To evaluate the risk of seizure recurrence and to guide patients with the wish to taper the ASM, risk calculators can be a valuable option, however, these calculators were not specifically designed for epilepsy patients with brain tumors [76]. These tools are on the one hand helpful for physicians to determine the recurrence risk in individual patients and on the other hand the visualization of a concrete percentage of risk of seizure recurrence can sometimes help to convince patients, who are not candidates for ASM withdrawal, to continue the medication.

In a prospective study on 83 patients with low grade or anaplastic glioma (of which 71 could be analyzed), who had been seizure free ≥ 1 year since last antitumor treatment or ≥ 2 years since the last seizure, a seizure recurrence in 26% (12/46) of patients who withdrew ASM and 8% (2/25) of patients who continued ASM was reported [77].

ASM should not be withdrawn in patients with progressive tumor, should not be stopped in patients with highly malignant tumors and short life expectancy and in those with difficult to control seizures in the past. In view of these authors, withdrawal of ASM is problematic in patients with little or no side effects of AEDs, with good social and professional reintegration after brain tumor therapy but with a high burden of social disadvantages if seizures recur.

ASM withdrawal is not recommended in patients with high risk of seizure recurrence independent of the duration of seizure freedom, however, determining the risk of seizure recurrence is difficult as it is influenced by multiple factors (e.g., tumor grade, location, type of treatment). In patients with grade 2 or 3 gliomas with a molecular profile that predicts favorable prognosis, stable disease of the tumor and long-term seizure freedom, tapering the medication might be considered in the following circumstances: if patients suffer from severe side effects (not solvable by changing the medication) or if there is an explicit patient wish even after detailed information about the risk of seizure recurrence and the consequences for daily living (e.g., fitness to drive) [78].

### Fitness to drive in brain tumor patients with epilepsy

The fitness to drive in brain tumor patients can not only be impaired by epilepsy but also by motor, sensory and coordination deficits, impaired vision, and neurocognitive deficits.

The regulations if and when patients with brain tumors, particularly in case of BTRE, are able to resume driving are complex and guidelines differ substantially between countries. In Germany, the Ministry for Transport and Digital Infrastructure (Bundesanstalt für Straßenwesen, BASt) publishes guidelines for the assessment of the ability to drive. These guidelines do currently (version of December 31, 2019) include no chapter that focuses explicitly on brain tumor patients. For patients who have undergone brain surgery, the German guidelines prohibit driving for three months after surgery [79]. For patients with epilepsy driving is prohibited for 12 months after the last seizure. For patients with BTRE both prohibitions apply (and both together determine the duration of the inability to drive). The prerequisite to allow driving again is a regular follow-up depending on the underlying tumor (e.g., three months in patients with glioblastomas, 6 months in patients with diffuse low-grade gliomas) [79]. In Switzerland a consensus paper has been published in 2021 requiring the following examinations every three months to acquire or to maintain fitness to drive for glioblastoma patients: cranial magnetic resonance imaging (MRI) according to Response Assessment in Neuro-Oncology (RANO) criteria, medical history and comprehensive neurological examination (optional plus electroencephalography), optional ophthalmological examination including examination of visual field, optional neuropsychological assessment (with “optional” meaning, if careful assessment of history and/or neurological examination gives hint to pathology in this respect) [80]. In patients with brain metastases a clinical neurological examination had shown very low sensitivity to predict fitness to drive in 41 patients, who subsequently underwent a standardized assessment (occupational therapy driving assessment). The authors assumed that this was due to the fact that neurological examination can only limitedly or not predict judgment, reaction speed and complex visual-motor functions [81].

In summary, to evaluate the fitness to drive again for brain tumor patients is a major challenge for clinicians demanding for a multidisciplinary approach, involving neurologists, radiologists, ophthalmologists and neuropsychologists. However, the prohibition or permission to drive is one of the crucial factors of participation in daily living and the evaluation can be worth the effort in appropriate candidates (i.e., stable brain tumor, long-term seizure freedom).

## Conclusions

Many patients with brain tumors suffer from epilepsy and treatment of BTRE is challenging. The evolving knowledge about the pathophysiological aspects of BTRE might influence future therapeutic recommendations. Current recommendations for ASM are based mostly on therapeutic recommendation in focal epilepsies in general, even though many trials on ASM excluded patients with brain tumors [47, 82]. LEV, LTG and LCM are non-enzyme inducing ASMs frequently used in BTRE. Randomized trials with matched patients concerning tumor type and additional medication, with reliable endpoints and careful assessment of side effects and quality of life are needed to determine optimal management of BTRE. Seizure freedom and ≥ 50% seizure reduction rate are commonly used endpoints in epilepsy trials, however, patients with brain tumors often have cognitive impairment preventing them to reliably report on their seizure frequency. Thus, ASM retention might be a more reliable endpoint with regard to therapeutic efficacy and is easily and routinely documentable during neurooncological follow-up [83]. ASM withdrawal and fitness to drive are aspects that require thorough work up before clinical decision making. Combined neurooncological end epileptological expertise is required to make decisions in these clinical situations.

Individualization of treatment approaches is increasingly addressed in both fields, epilepsy and neuro-oncology. Thus, the ideal future of BTRE treatment might comprise individualized antiseizure medication for different tumor types and stages with an additional effect on brain tumor proliferation. If perampanel might be such a drug needs to be evaluated in future studies.

## Data Availability

Data sharing is not applicable to this article as no datasets were generated or analysed during the current study.

## Abbreviations

ASM: Antiseizure medication
AMPA: α-Amino-3-hydroxy-5-methyl-4-isoxazolepropionic acid
AMPAR: α-Amino-3-hydroxy-5-methyl-4-isoxazolepropionic acid receptor
BTRE: Brain tumor related epilepsy
GBP: Gabapentine
IDH: Isocitrate dehydrogenase
ILAE: International league against epilepsy
LCM: Lacosamide
LEV: Levetiracetam
LTG: Lamotrigine
MRI: Magnet resonance imaging
OS: Overall survival
RANO: Response assessment in neurooncology
SE: Status epilepticus
TPM: Topiramate
VPA: Vaproic acid
ZNS: Zonisamide
